# “A Dying Cause”—Vivisection Fallacies*From Editorial in Life.

**Published:** 1911-05-15

**Authors:** J. U. Lloyd


### "A DYING CAUSE"*—VIVISECTION FALLACIES.

BY J. U. LLOYD, M.D.

The record of past events demonstrates that it is useless, as a rule, to attempt to counteract a fallacy until it has run its course. Some people must, by their own personal experience, prove that there is "nothing in it." Only when they do this, and become tired of the fad, are they ready to divert themselves by the taking up of another.

In 1885 this writer demonstrated to his own satisfaction that vivisection was a cruel but yet a coming fad. He furthermore accepted that, owing to the conspicuity of the leaders in that direction, its followers must increase like a flock of locusts until the pest should run its course. Some day, possibly, a yet unpublished chapter of Etidorpah

*From Editorial in Life.

may be printed, in which, fifteen or more years ago, the present vivisection fad was graphically portrayed. The chapter was reluctantly suppressed, through the request of Professor W. H. Venable, who argued that, although probably true to fact, the subject was so horrible, as presented as to render it too excruciatingly painful for a work designed for general circulation. It is not our purpose, however, to touch upon that phase of the subject in the present article, our object now being to point out a few views that this writer yet holds concerning the establishing of the value of a remedy in disease by its power of injuring or killing creatures in health.

In this, however, it seems as though balanced thinkers, involved in the direction of the vivisection fad, are beginning to ponder over the record of it all. For example, let us reproduce the following, as reported by an Associated Press dispatch:

"Twenty-nine years' practice as surgeon to the London Cancer Hospital so thoroughly convinced Dr. Herbert Snow, of London, that 'what passes with the public as cancer research is utter moonshine,' that he stirred the members of the Anti-Vivisection Society meeting here last night with a condemnation of 'the whole system of experimentation upon the sub-human animals.' As an instance he pointed out that lemonade, a healthful beverage for man, was deadly poison to cats and rabbits. 'Salt', he said, 'is fatal to chickens; prussic acid promptly kills men and elephants, but horses and hyenas take it with impunity. Rabbits eat belladonna, goats are fond of the tobacco plant and of conium, the hemlock which killed Socrates. And so one sees the mischievous character of drug experiments on the lower animals.

Let us add a few thoughts in the direction of paralleling the above exceptions to the fallacy that healthy lower animals can be utilized to demonstrate the action of a drug upon man, either in health or sickness.

The hog can eat poisons, as arsenic, that will kill a dog.

And yet hogs have been killed by too much sweet milk. Shall we then feed babies with arsenic instead of milk? The hog likewise can withstand the poisons of venomus creatures, and in some sections of Africa, where terribly poisonous serpents abound, a drove of hogs is made to serve as an advance guard through a jungle. "Chambers' Miscellany" reports that the hog presents one cheek to the poisonous reptile, which fastens its fangs therein, thus giving to the hog the chance to shred the reptile with his feet and teeth, without, however, experiencing any ill effects from the poisonous bite. Snake-bites are therefore proven good for sick men!

The rat, that distributes the bubonic plague, is practically immune to the poison of the disease. The flea, that bites the rat and then bites man, is not affected by the poison that kills the man. Consequently, bubonic plague germs are "scientifically" harmless to humanity. The mosquito that distributes the typhoid germ as well as that of the yellow fever, sings as it passes through the air with its load of deadly poison. Therefore neither of these diseases are harmful to mankind.

Insects there are that live in cayenne pepper and thrive on this most energetic substance. Others thread themselves through and through the strongest tobacco. which in turn poisons or is harmful to the majority of insects. Do these facts prove that nicotine is not a poison and must not be considered seriously? "Chambers' Miscellany" reports that during the siege of Moscow the bullets of the French soldiers were honeycombed by insects that had eaten the lead with impunity. Consequently lead is harmless to mankind! The deadly nightshade is a tasteful morsel to certain insects related to the well- known cantharides, and under the onslaughts of these, every vestige of green on the plant may disappear in a single day or night. Belladonna is therefore an excellent food for man. The "tumble bug" thrives in manure and the buzzard lives on carrion. Let men cease to fear filth and turn to such food as this.

The dog, so incessantly murdered by vivisection enthusiasts, is sensitive to a touch of strychnine that will not (it is reported) affect a hawk, but will yet kill a chicken. It is thus wise to feed strychnine to people. A few grains of salt, scattered on our lawn by accident, killed an entire flock of beautiful "wax wing" birds. Salt is therefore a poison to mankind, and must not be used in food.

We once knew morphine injected into a rabbit's veins sufficient in amount to kill two men, but without any sign of sleepiness on the part of the rabbit. Consequently morphine will not produce sleep in man.

In this direction we quote from a paper on "Immunity," printed in the "Proceedings of the Missouri Pharmaceutical Association," 1902, by Mr. J. F. Llewellyn, of Mexico, Mo.:

"Dogs and horses, in proportion to weight, endure ten times as much morphine as men; doves five hundred, frogs one thousand times as much. The fatal dose of calomel for a cow is the same as for a hog. One pound of sugar of lead is required to kill a horse, while one-tenth will kill a cow of the same weight.

"The hedgehog is immune to all poisons, the mongoose to cobra poison, snakes generally to their own poison, the king snake to all snake poisons.

"Goats eat tobacco, while tall larkspur kills cattle. Lupine hay kills sheep, but horses are immune. Aconite kills sheep, horses and goats being immune. Hyoscyamus kills men and rabbits; cattle, sheep and goats are immune. Antimony, that kills men and most animals, will not harm hogs or elephants.

"Acquired immunity is as difficult a problem as natural. Of strychnine, Jack used five to ten grains at a dose. Arsenic eaters of Styria reach fourteen grains at a dose. A man of seventy had used it for forty years, while a patient in Vienna used twenty-two grammes yearly with good results.

"In northern China all smoking tobacco is mixed with arsenic, and the smokers are stout fellows, with lungs like bellows. In like manner, sixty-four ounces of laudanum has been used by a fiend monthly.

"Sheep acquire a loco habit, teach other sheep its use, and prefer it as food.

"The dried poison gland of the cobra, eaten, gives immunity to cobra poison. It has an intoxicating effect, not impaired by habitual use. Pigeons with gradually increased doses of cobra poison, survived seven times the lethal dose."

The foregoing statements, however, may be resisted by vivisection faddists on the ground that the physiological investigator injects his poison (or what not) into the *blood* of the creature, determining the effect the same substance will have when taken internally or hypodermically by a human being in sickness. To be just, let us then argue that the method of determining the ammount of lead a rabbit could take into its stomach without endangering its health, if normal, or to cure it if sick, is to be decided by studying the amount of shot required to kill a squirrel by means of a shotgun. Shoot a squirrel full of shot, and determine therefrom the action of lead taken into the stomach of an ailing rabbit.—*Electic Medical Journal.*

